# Retraction: Role of Statins in Reducing Cardiovascular Mortality: A Systematic Review of Long-Term Outcomes

**DOI:** 10.7759/cureus.r171

**Published:** 2025-04-01

**Authors:** Bipasha Seth, David Okello, Syed Saad Ullah, Rabia Yousaf, Shaikha B Alfalasi, Muhammad Hafeez, Naveed Rasool, Gurman Bhullar, Tchapleu Nana Ian Gidley, Syed Ali Hussein Abdi, Khakan Murtaza

**Affiliations:** 1 Intensive Care Unit, Niraj Intensive and Anesthesia Care Private Limited, Delhi, IND; 2 Internal Medicine, Ministry of Health, Lusaka, ZMB; 3 Pulmonology, Jinnah Postgraduate Medical Center, Karachi, PAK; 4 Internal Medicine, Shifa College of Medicine, Islamabad, PAK; 5 General Surgery, Dubai Medical College for Girls, Dubai, ARE; 6 Pharmacology, Quetta Institute of Medical Sciences, Quetta, PAK; 7 Internal Medicine, East and North Hertfordshire NHS Trust, London, GBR; 8 Internal Medicine, Sri Guru Ram Das University of Health Sciences and Research, Amritsar, IND; 9 Internal Medicine, Faculty of Medicine and Biomedical Sciences, Yaoundé, CMR; 10 Internal Medicine, Ajman University College of Medicine, Dubai, ARE; 11 Internal Medicine, Nishtar Medical University, Multan, PAK

The Editors-in-Chief have retracted this article. An investigation by the journal found evidence of authorship manipulation. The Editors-in-Chief therefore no longer have confidence in the provenance and reliability of the article contents. The authors agree with the decision to retract.

